# Supplementary material for: Interplay of hot electrons from localized and propagating plasmons
Source: Nat Commun. 2017 Oct 3;8:771. doi: 10.1038/s41467-017-00815-x (PMC5626744; doi:10.1038/s41467-017-00815-x)
Supplement: Supplementary file 1 — Supplementary Information [file 41467_2017_815_MOESM1_ESM.pdf]

SUPPLEMENTARY FIGURES

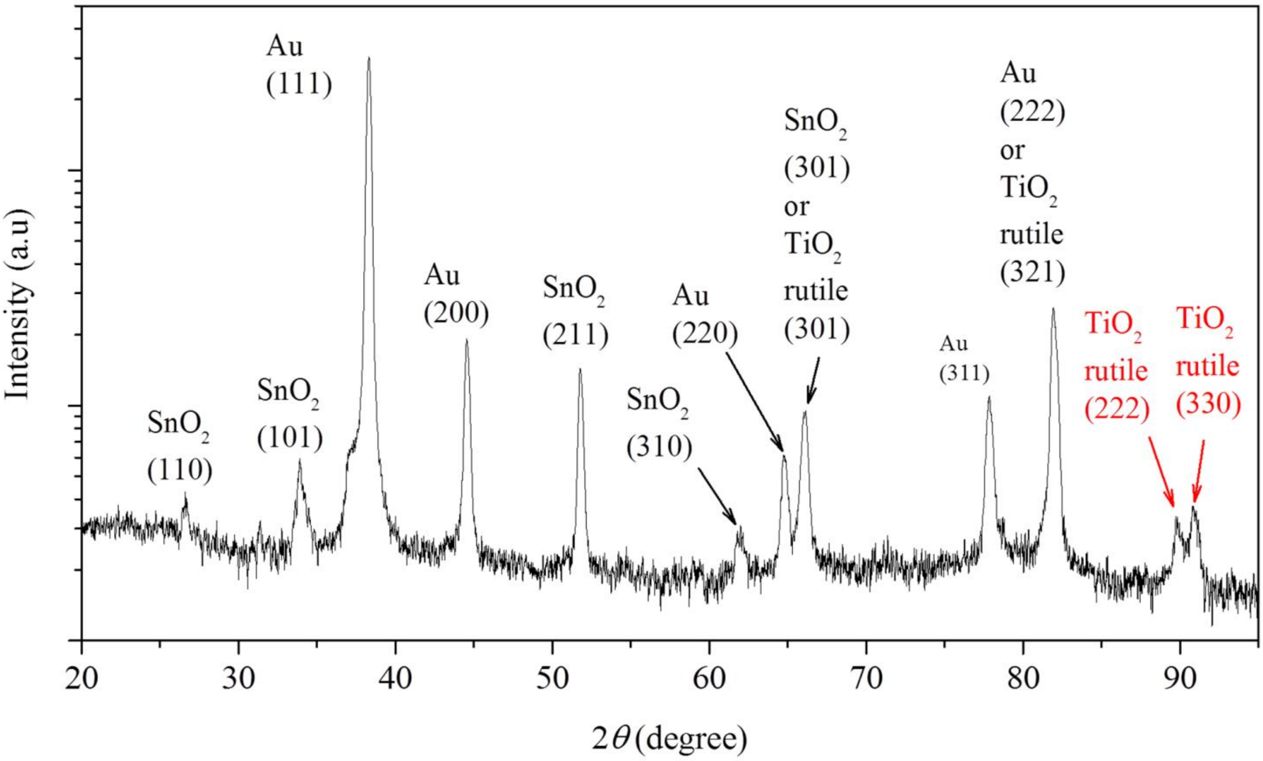

Supplementary Figure 1 | X-ray diffraction pattern of the TiO_2 layer prepared on an Au/FTO/glass substrate. The diffraction patterns of the Au film and SnO_2 are clearly evident. The rutile phase patterns of TiO_2(321) and TiO_2(301) are superimposed with those of Au(222) and SnO_2(301). Also, TiO_2(222) and TiO_2(330) are clearly evident (indicated by the red arrows).

a

b

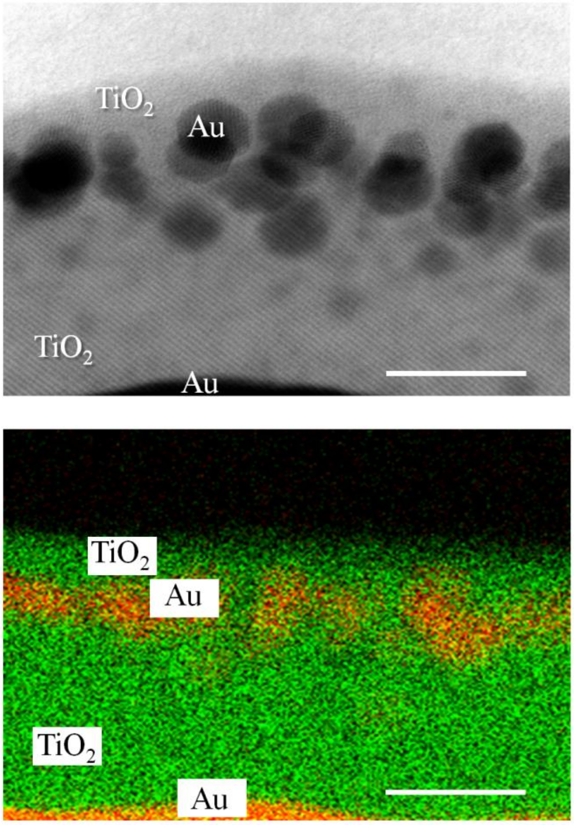

Supplementary Figure 2 | Transmission electron microscope (TEM) image and element map of the dual-plasmon device. **a**, Cross-sectional TEM image of the device. **b**, Cross-sectional element map of the device using the TEM with energy dispersive analysis. The areas of green and orange colors represent the presence of the TiO_2 and Au material, respectively. Scale bars are 5 nm for both cases.

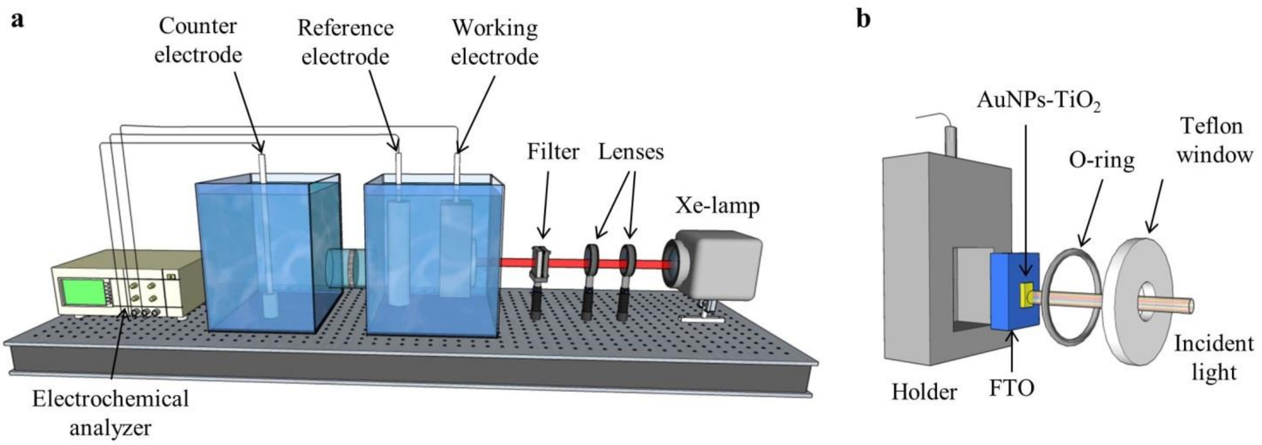

Supplementary Figure 3 | Schematic of the photoelectrochemical measurement setup. a, Optical configuration of the setup. **b**, Sample holder for supportof the dual-plasmon device as the working electrode in the photoelectrochemical measurement.

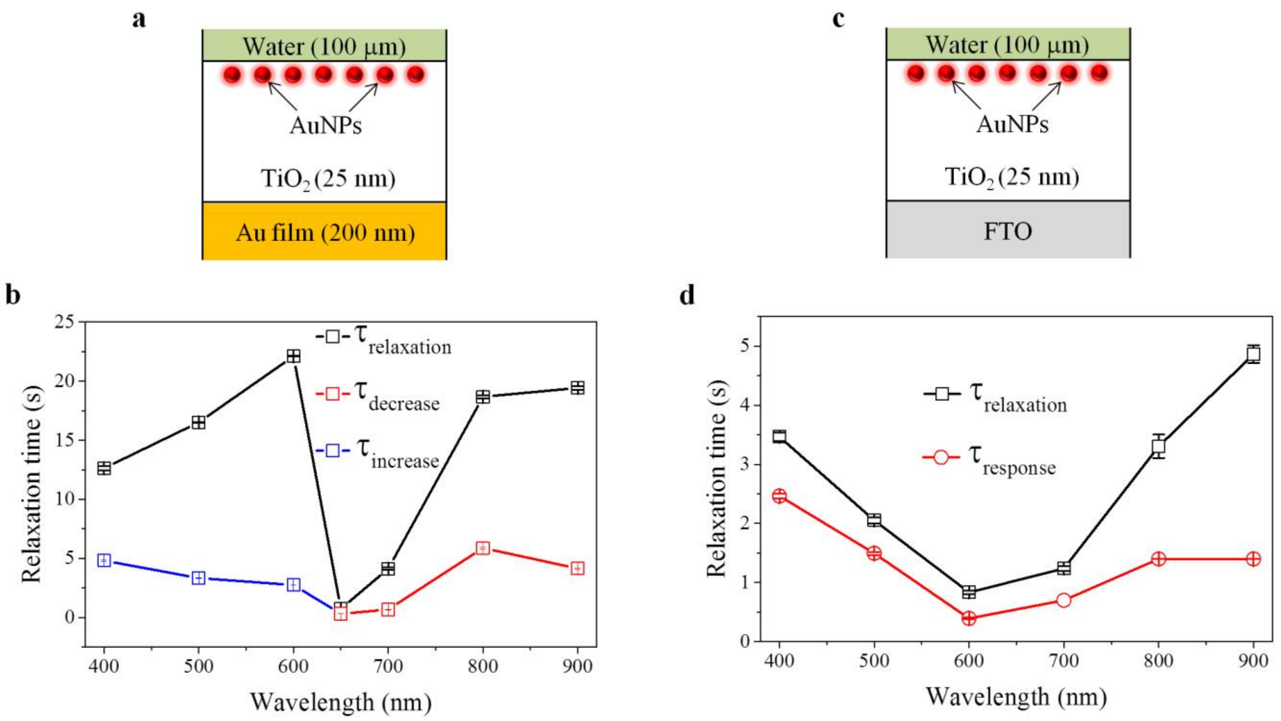

Supplementary Figure 4 | Response and relaxation of the electron transfer. a, Schematic of the dual-plasmon device. **b**, Response time and relaxation time of the measured photovoltage at different illumination wavelengths. **c**, Schematic of the device without the Au film. **d**, Response time and relaxation time of the measured photovoltage at different illumination wavelengths.

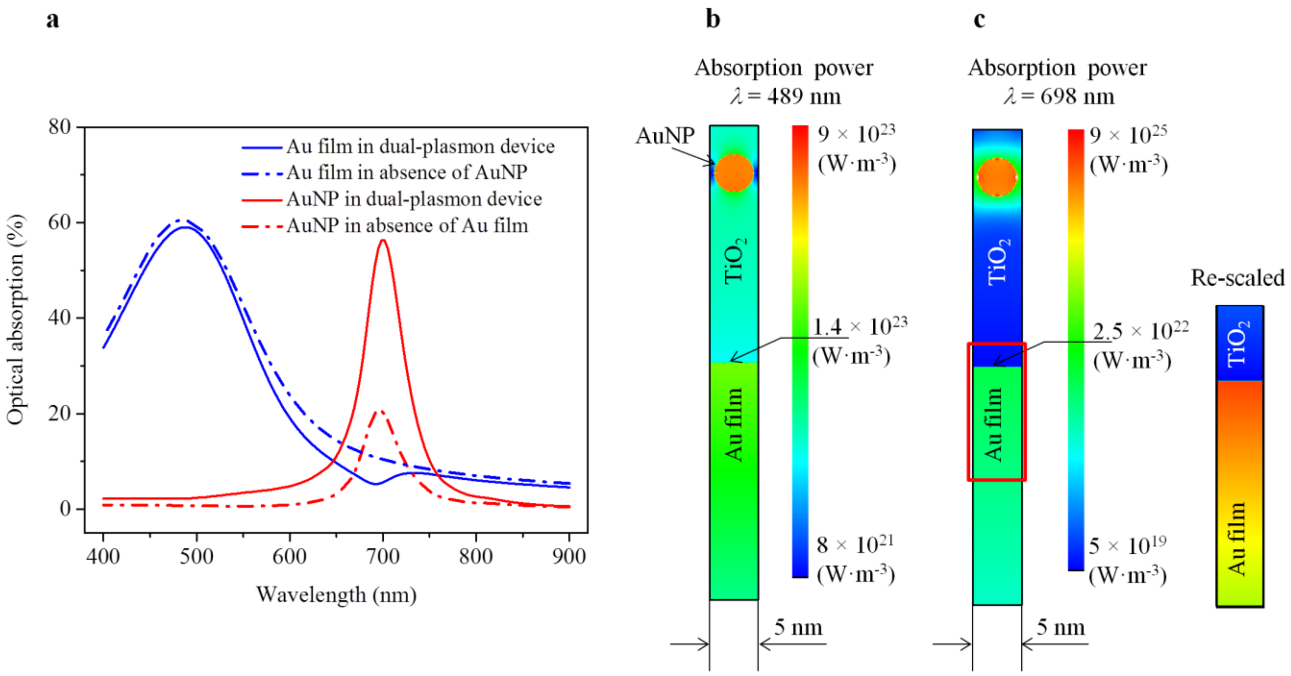

Supplementary Figure 5 | Numerical simulations. a, Optical absorption on the Au film and AuNP in the dual-plasmon device (solid curves), and in the device without the AuNP or Au film (dash-dotted curves). **b**, Absorption profile of the dual-plasmon device at an illumination wavelength of 489 nm (off-resonance wavelength with the LSPR of the AuNP). **c**, Absorption profile of the dual-plasmon device at an illumination wavelength of 698 nm (on-resonance wavelength with the LSPR of the AuNP). The inset indicates the absorption profile at the Au film/TiO_2 layer interface with a re-scaled color set.

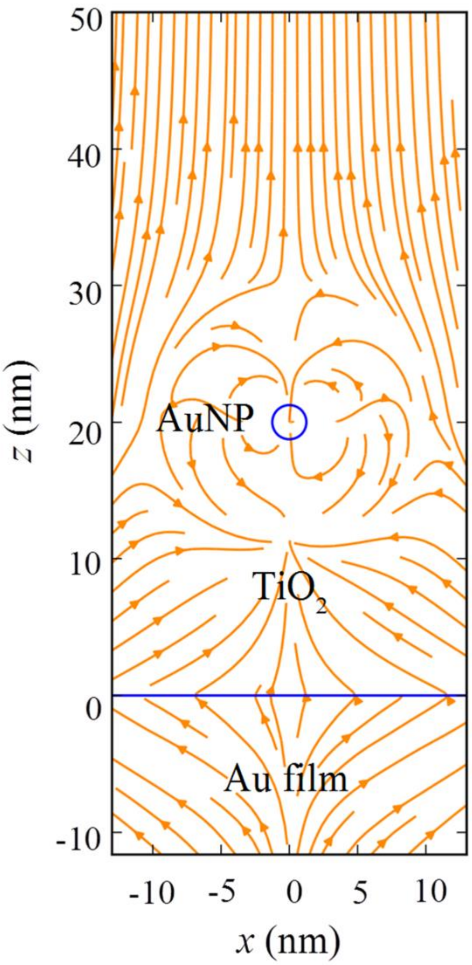

Supplementary Figure 6 | Finite different time domain (FDTD) simulation of the power flow in the device. It is presented as a snapshot at 1.8 fs after the incident light hits the device. The direction of power flow in the device is indicated by the direction of the Poynting vectors. For clarity, the boundaries of the Au cluster/TiO_2 and Au film/TiO_2 layers are highlighted in blue.

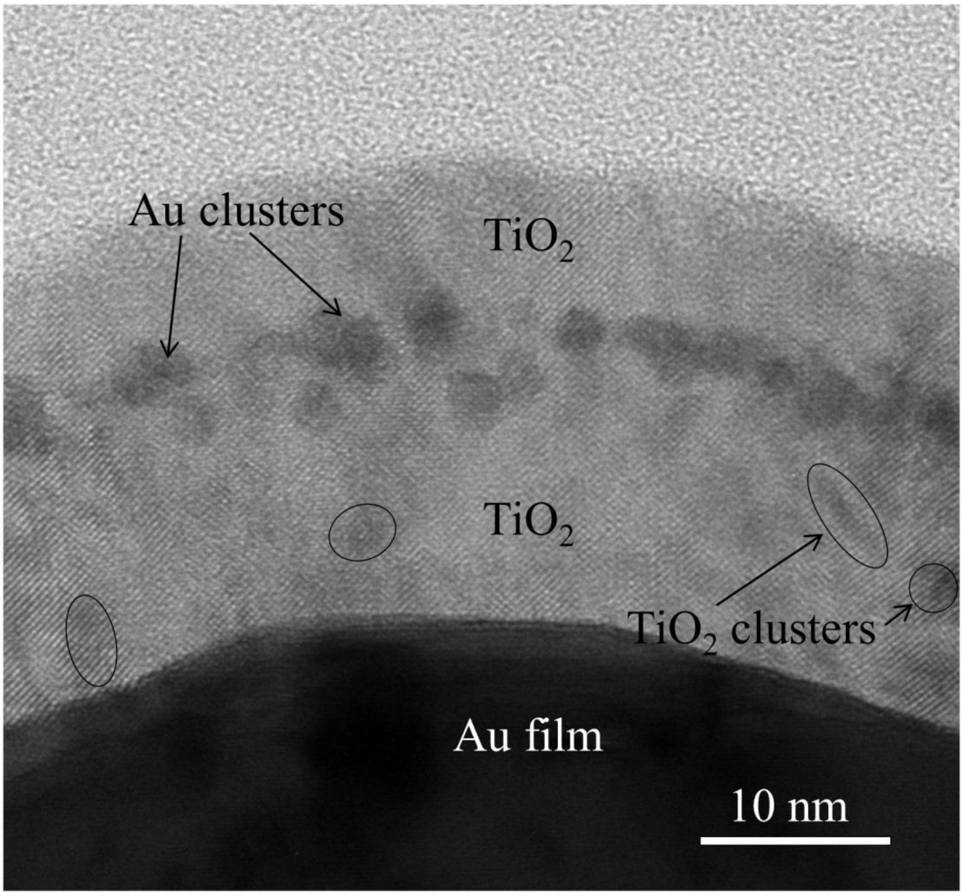

Supplementary Figure 7 | Transmission electron microscope (TEM) image of the Au clusters. The spherical Au clusters are formed by depositing a 0.3 nm thick Au layer in a TiO_2 layer and subsequent annealing. The Au clusters have an average diameter of about 2.5 nm. The small TiO_2 nanoclusters and particles are marked with black ellipses.

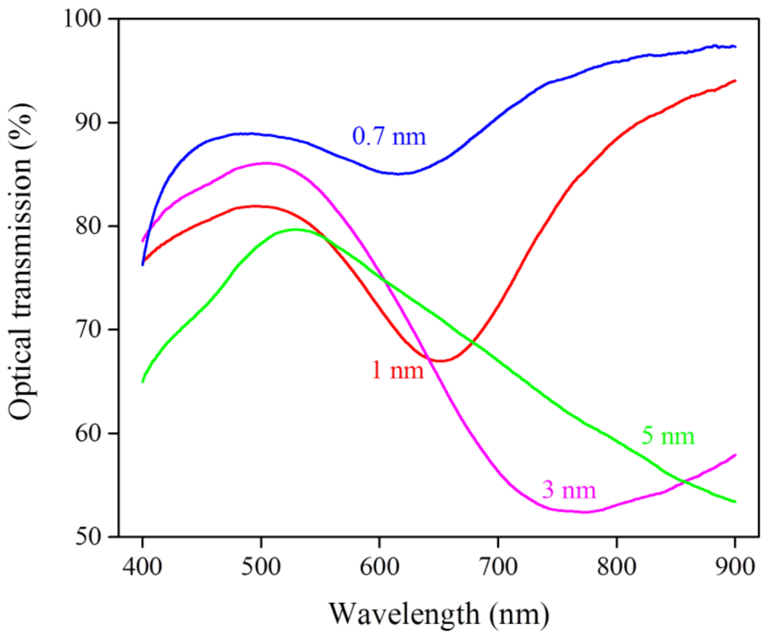

Supplementary Figure 8 | Tailoring the localized surface plasmon resonance (LSPR) of the AuNPs by varying the thickness of the thin Au layer before the annealing process to form the AuNPs. Shown in the figure is the optical transmission through the device in the absence of the 200 nm thick Au film at various thickness values for the thin Au layer.

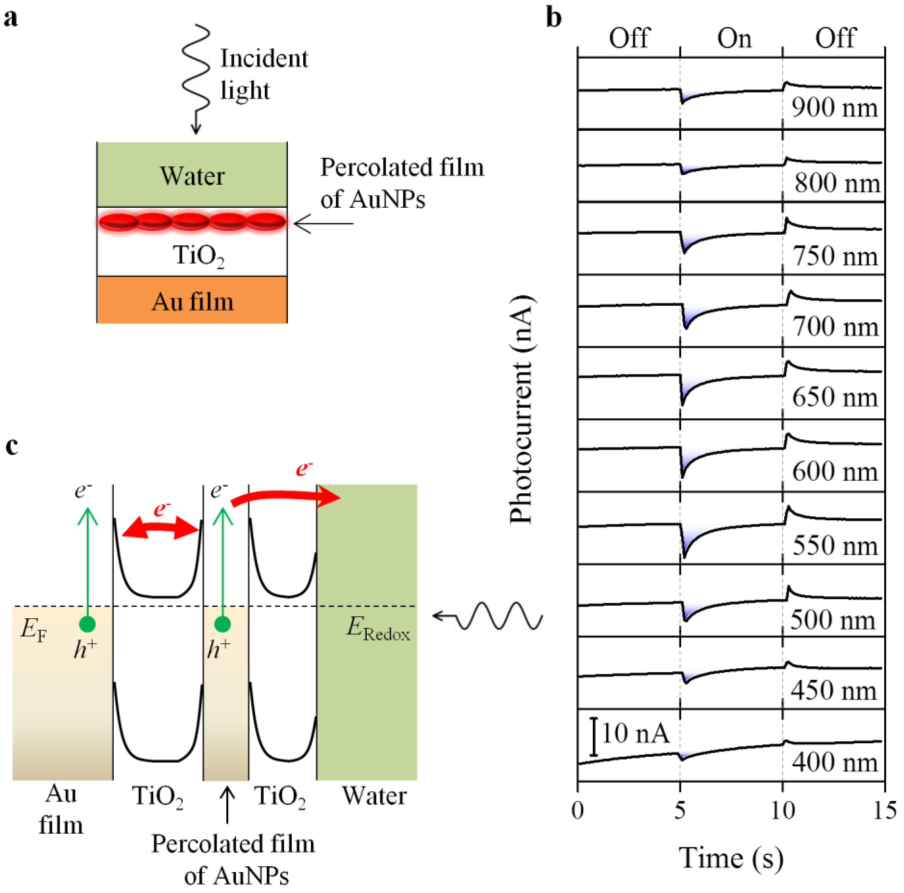

Supplementary Figure 9 | Absence of the interplay effect in the device with a percolate film of AuNPs. a, Schematic of the device. **b**, Results of the photocurrent measurements. **c**, Band diagram showing the generation and transfer of hot electrons in the device.

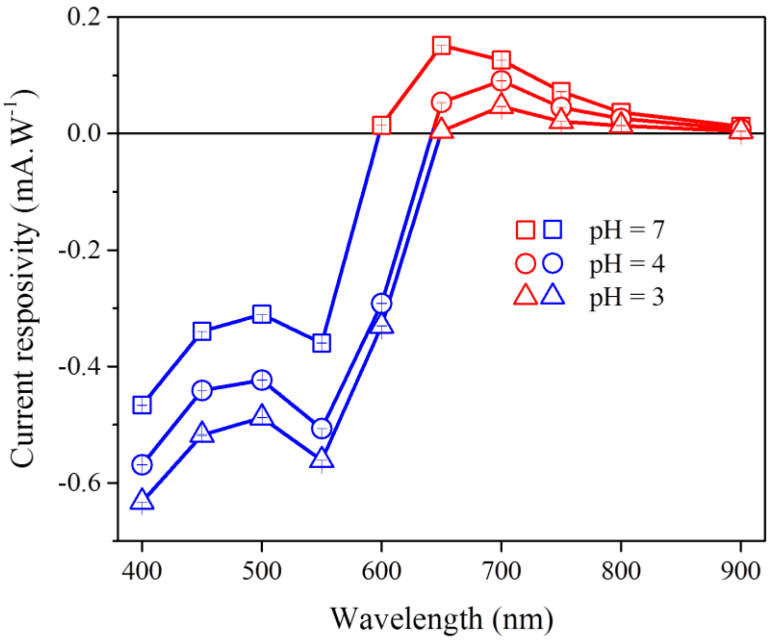

Supplementary Figure 10 | Current responsivity of the dual-plasmon device for aqueous solutions at various pH values. The photocurrent measurements are performed under three different pH conditions (pH = 3, 4, 7).

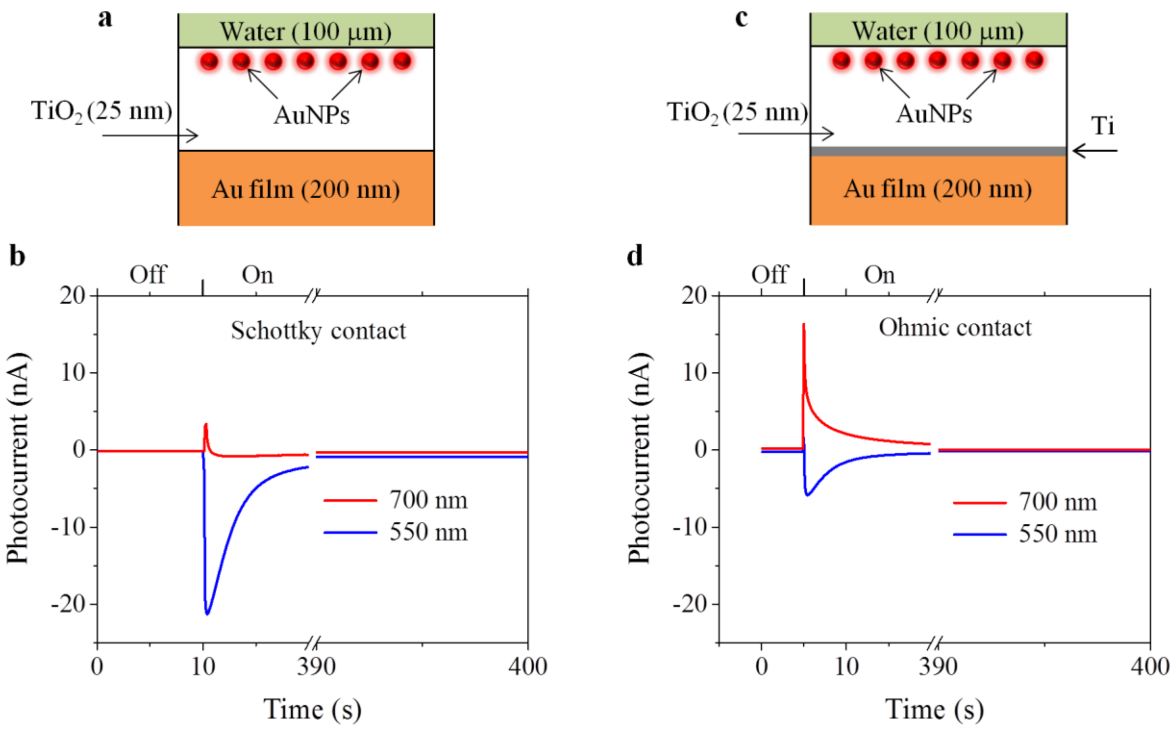

Supplementary Figure 11 | Comparison of the Schottky and Ohmic contacts in the performance of the dual-plasmon device. **a**, Schematic of the device withSchottky contact. **b**, Results of the photocurrent measurements at illumination wavelengths of 550 nm and 700 nm. **c**, Schematic of the device with the Ohmic contact. **d**, Results of the photocurrent measurements at illumination wavelengths of 550 nm and 700 nm. The presence of a thin Ti layer between the Au film and TiO_2 layer enables the formation of the Ohmic contact. For both devices, the LSPR wavelength of the AuNPs is about 650 nm.

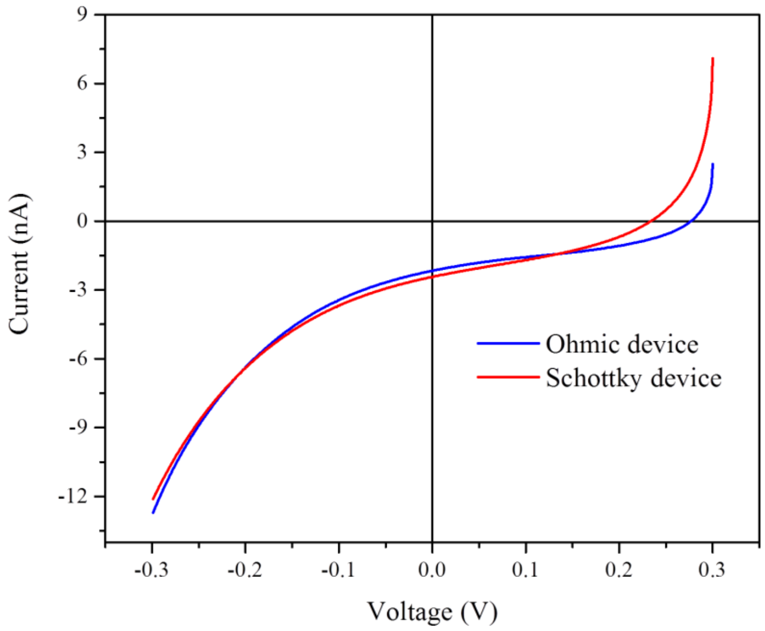

Supplementary Figure 12 | Comparison ofSchottky and Ohmic contacts in regard to the performance of the dual-plasmon device. The current-voltage measurements are performed under non-illumination conditions on the devices withSchottky- and Ohmic contact.

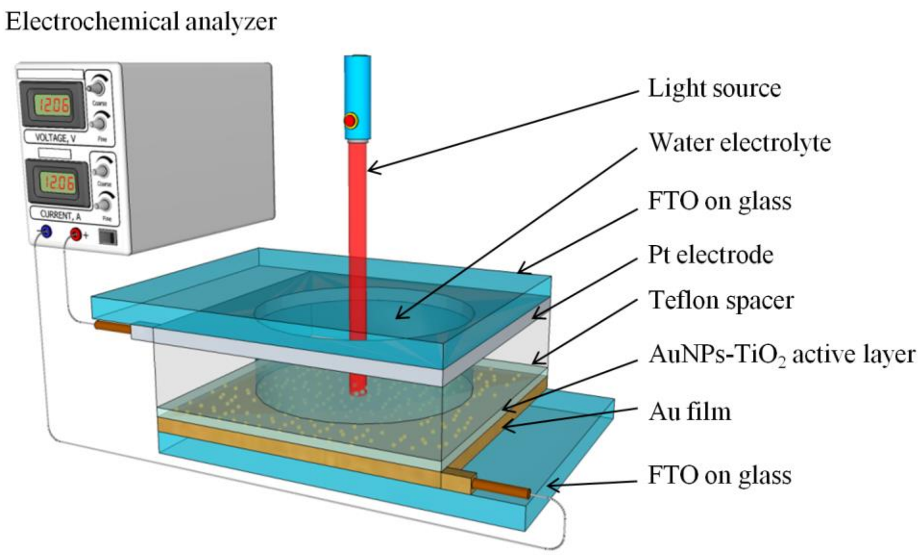

Supplementary Figure 13 | Schematic of the demonstration of the wavelength-controlled polarity-switchable photoconductance. The polarity-switching wavelength of the dual-plasmon device is about 600 nm. The illumination is conducted by using two laser diodes at 535 nm and 650 nm. A Movie of the demonstration is available as Supplementary Movie 2.

SUPPLEMENTARY NOTES

Supplementary Note 1. Animated Movie showing the functionality of the dual-plasmon device. The Movie shows two representative cases that correspond to those shown in Figure 2. Since the direct generation of hot electrons by inter-intraband transitions is common for both cases, it is neglected in the Movie. In the first case, the dual-plasmon device is excited by the incident light whose wavelength is off the LSPR of the AuNP (denoted by the blue arrow). The incident light is Rayleigh-scattered by the AuNP and impinges on the Au film at large incident angles exciting random SPPs at the interface between the Au film and TiO_2 film (depicted by the change in the color of the Au film from yellow to red). The SPPs propagate along the interface and generate hot electrons by non-radiative plasmon decay. At the same time, the AuNP excited by the incident light generates a small number of hot electrons by non-radiative plasmon decay. The net photocurrent is negative. In the second case, the dual-plasmon device is illuminated by the incident light whose wavelength is on the LSPR of AuNP (denoted by the red arrow). Similar to the first case, random SPPs are generated and decay by producing hot electrons. At the same time, the plasmonically resonant AuNP (depicted by the change in the color of the AuNP from gray to red) emits a large number of hot electrons by non-radiative plasmon decay. Relative to the water layer, the majority of the generated hot electrons from the AuNP is directed toward the Au film. Also, the AuNP emits omnidirectional light by radiative plasmon decay, inducing another SPP. The net photocurrent is positive.

Supplementary Note 2. Demonstration of the wavelength-controlled polarity-switchable photoconductance. The Movie shows the experimental demonstration of two representative cases that correspond to those shown in Figure 2. A schematic of the demonstration is shown in Supplementary Figure 13. The dual-plasmon device is fabricated aiming at having the polarity-switching wavelength located between the wavelengths of two laser diodes for the demonstration: 535 nm (green) and 650 nm (red). For this purpose, a 1 nm thick Au layer is deposited in the TiO_2 layer and followed by an annealing step to produce AuNPs. The fabricated dual-plasmon device shows a LSPR peak at ~650 nm and exhibits a polarity switch at ~550 nm under zero bias condition. For a clear experimental demonstration of wavelength-controlled polarity-switchable photoconductance, the device is negatively biased at -0.1 V between the Pt electrode and the active layer to tune the polarity-switchable wavelength to a value between 535 nm and 650 nm. The photocurrent readout is recorded by an electrochemical analyzer (scaled between -10 V to +10 V)

and is sent to a home-built electrical circuit and used to control the lighting condition of the laser diodes.

Supplementary Note 3: Photocurrent and photovoltage measurements

As shown in Supplementary Figure 3a and Supplementary Figure 3b, the functionality of the dual-plasmon device was investigated by the conventional photoelectrochemical (PEC) measurement technique (electrochemical analyzer, model 802D, CH Instruments, Inc.) in a three-electrode configuration: Pt as a counter electrode, Ag/AgCl as a reference electrode, and the dual-plasmon device as a working electrode. In this configuration, a custom-made PEC cell was constructed in a single Pyrex glass reactor compartment (~80 % of optical transmission at λ = 350 nm and > 90 % of optical transmission from 400 nm to 900 nm) with a salt bridge at the middle. A 0.1M $KClO_4$ (from Kanto Chemical Co. Ltd.,) with a pH of ~7 was used as electrolyte solution. The optical illumination was performed with a Xe lamp and various cut filters (with a bandwidth of 10 nm) from the visible to near-infrared band. The illumination area on the working electrode was fixed to S = 3.14 mm^2. In this configuration, the applied voltage is located between the counter and reference electrodes. To convert the potential between the working and reference electrodes to the reversible hydrogen electrode (RHE), Nernst's equation can be used as follows: $V_{\mathrm{RHE}} = V_{\mathrm{Ag/AgCl}} + V_{\mathrm{Ag/AgCl-RHE}} +$ 59 × pH (mV). At pH = 7, $V_{\mathrm{Ag/AgCl-RHE}}$ = 197 mV at room temperature. Therefore, $V_{\mathrm{RHE}} = V_{\mathrm{Ag/AgCl}} +$ 610 mV.

Supplementary Note 4: Analysis of the time-varying photovoltage

Measuring and evaluating the evolution of a time-varying photovoltage is a useful technique to study the transport of charges in photoelectrochemical devices [1, 2]. The evolution of photovoltage can be analyzed by using the equation,

$$V(t) = V_0 + Ae^{-t/\tau}, \qquad (1)$$

where V_0 is the offset voltage, A is a constant, and τ is the time constant that defines the decay of the photovoltage which is subject to fitting. The resulting total half life given by $\tau \ln 2$ represents the total electron transfer process (including electrons trapped by defects in TiO_2) and was used for the analysis in Supplementary Figure 4b and Supplementary Figure 4d where the half life as a function of the illumination wavelength was plotted. Specifically, Supplementary Figure 4b shows the response time (when the light is turned on) and relaxation time (when the light is turned off) of the

photovoltage at each illumination wavelength obtained by fitting exponential functions (Eq. 1) to the data shown in Figure 3b. The figure indicates that, at all wavelengths except for the wavelength of the polarity switch, the transfer of the electrons from the Au film to the water is almost wavelength-independent whereas the transport of the electrons from the water to the Au film undergoes a sharp increase at the polarity switch wavelength and near the LSPR wavelength of the AuNPs. This is because the non-radiative decay of the localized plasmons in the AuNPs causes the emission of hot electrons with the highest efficiency at the LSPR wavelength. A similar behavior has also become evident in the device without Au film, as shown in Supplementary Figure 4d. Here, the response and relaxation times were obtained by fitting Eq. 1 to the measured photovoltages presented in Figure 5c. In comparison to the dual-plasmon device, the device without Au film provides faster electron transfer processes with and without the optical illumination. This is because the Schottky barrier between the Au film and TiO_2 layer in the dual-plasmon device acts as a barrier to electron transfer, making the process analogous to that of a capacitor and resulting in the formation of the burst-like photocurrent waveform at illumination wavelengths shorter than 650 nm.

Supplementary Note 5: FDTD simulations

The FDTD simulations were performed with FDTD Solutions (Lumerical, Inc.) to numerically calculate the 3D optical absorption profiles of the AuNP and Au film. The dielectric functions of Au and TiO_2 were obtained from Ref. [3, 4]. Specifically, the FDTD simulations were performed on a discrete, non-uniformly spaced mesh with the resolution ranging from 0.05 nm to 10 nm in a volume of 14 nm (x) × 14 nm (y) × 340 nm (z). A forced uniform mesh with the resolution of 0.05 nm was added to the AuNP in the volume (5 × 5 × 5 nm^3). For the FDTD calculation of the randomly distributed AuNPs (presented in Figure 3f), 50 AuNPs of different particle sizes ranging from 3.5 nm to 4.5 nm with random distribution (laterally) on the TiO_2 layer were used. The resolution of the calculation was 0.2 nm for the AuNPs and TiO_2 layer. In other volumes, the mesh remained non-uniform ranging from 0.2 nm to 10 nm. Periodic boundary conditions were used in both the x and y directions. In the propagation direction of incident light (z direction), perfectly-matched-layer-boundary conditions were applied. The linearly polarized plane wave was incident onto the x-y plane at normal incidence. Supplementary Figure 5a shows the optical absorption profile evaluated over the whole volume of the Au film (14 × 14 × 200 nm^3) and in the volume (5 × 5 × 5 nm^3) containing the AuNP in the device without the AuNP or Au film. The figure indicates that, by introducing the Au film, the absorption of the AuNP increases dramatically. The LSPR wavelength of the AuNP slightly shifts from 693 nm to 698 nm in the presence of the Au film. This small shift can be

understood in terms of the electromagnetic coupling of the AuNP and Au film. Since the separation between AuNP and Au film is 20 nm, which is large for the near-field coupling, a small red-shift is expected and accounts for the shift [5]. The drastic increase in the absorption of the AuNP, due to the presence of the Au film, reflects the importance of this design in maximizing the harvesting efficiency of incident light. Supplementary Figure 5b and Supplementary Figure 5c show the absorption profiles of the dual-plasmon device illuminated at two different excitation wavelengths of λ = 489 nm (off-resonance with the LSPR of the AuNP) and λ = 698 nm (on-resonance with the LSPR of the AuNP). At the on-resonance with the LSPR, the AuNP displays an extraordinarily large optical absorption (by two orders of magnitude) compared to that at the off resonance with the LSPR. On the contrary, the off-resonance with the LSPR excitation provides nearly one order of magnitude higher power density in the Au film than the on-resonance with the LSPR excitation due to the interband transition in the Au film.

An adjusted color scale allows us to see the absorption at the Au film/TiO_2 layer interface, as shown in Supplementary Figure 5c (a similar result for Supplementary Figure 5b, not shown), since the color scale in these figures was originally set to emphasize the absorption intensity at the AuNPs at the expense of showing the absorption intensity in the Au film awhich made it difficult to see the evidence for the SPPs in the figure. Due to this adjustment, the color around the AuNP is oversaturated and not shown in the figure. Consequently, the absorption intensity is only evident at the Au film/TiO_2 layer interface after adjusting the color scale. In Supplementary Figure 5c, the absorption at the Au film/TiO_2 layer interface is higher than that in the surrounding area, indicating field enhancement at this interface. The reason for this field enhancement is eitherthe existence of the SPPs at the interface or the penetration of incident light to the Au film (here the penetration depth of the visible light in the Au film is ~20 nm [6]).

In order to clarify this point, we performed an additional FDTD simulation to investigate the power flow in the device. Supplementary Figure 6 presents the result of the simulation of a device consisting of a 2.5 nm diameter cluster embedded in the TiO_2 layer. The distance between the cluster and the Au film is 20 nm. The light source is a pulse-like plane wave normal to the Au film at a wavelength of 635 nm. The grid size is non-uniform, being 0.488 nm around the Au cluster and 2 nm in other areas. The dielectric function of the Au and TiO_2 is taken from literature [2, 3]. In Supplementary Figure 6,a simulation of the Poynting vectors in the device is presented. In the figure, the direction of the Poynting vectors is from the Au film towards the Au cluster as the incident light

is reflected by the Au film (after ~1.8 fs after the light hits the Au cluster). As the Poynting vectors reflect the power flow in the device, the non-uniformity of the Poynting vectors obtained in this simulation indicates the existence of the SPPs at the Au film/TiO_2 layer interface.

Supplementary Note 6: Tailoring the LSPR of the AuNPs and the effect of the device with a percolated film of AuNPs

Since the optical properties of NPs as optical antennas depend on their size, shape, and dielectric properties as well as the surrounding medium [7, 8], it is possible to tune the LSPR of the AuNPs by varying these parameters. Supplementary Figure 8 shows the tunability of the LSPR wavelength of the AuNPs by changing the thickness of the Au film embedded in the TiO_2 layer before annealing it, ranging from 0.7 nm to 5 nm. The optical measurements were performed in absence of the 200 nm thick Au film. As shown in the figure, the LSPR peak displays a redshift as the thickness of the Au film increases, since this corresponds to the increase in the particle diameter. Moreover, the figure also shows that the device with the 5 nm thick Au film does not show LSPR. This is due to the formation of a percolated film of AuNPs [9] as schematically shown in Supplementary Figure 9a. As presented in Supplementary Figure 9b, the device that consists of this percolated film of AuNPs exhibits only negative photocurrent for the entire spectral range. The working principle of this effect is schematically explained in Supplementary Figure 9c. Since the percolated Au film is expected to be composed of connected islands, the generation of hot electrons by LSPR does not occur. Hence, the direct excitation of hot electrons from the inter-intraband transitions induces a negative photocurrent from the percolated Au film to the water layer, similar to that of an observation reported in a recent report [9]. The percolated Au layer is semi-transparent such that the generation of hot electrons from the 200 nm thick Au film by direct optical excitation is possible. Therefore, a capacitor-like effect occurs between these two Au films, resulting in a charging-discharging effect as depicted in Supplementary Figure 8c.

Supplementary Note 7: Comparison of Schottky and Ohmic contacts in the performance of the dual-plasmon device

In order to elucidate the effect of the Schottky barrier on the photocurrentgeneration and polarity-switching phenomenon, we compared the device's electrical properties with and without the Schottky barrier between the Au film and TiO_2 layer (Supplementary Figure 11a, Supplementary Figure 11c). The Schottky barrier was eliminated (to form an Ohmic contact) by depositing an approximately 3 nm Ti layer between the Au film and the TiO_2 layer [10]. Specifically, the following steps were performed to synthesize the Ohmic contact: (1) sputtering a 200 nm thick Au film, (2)

sputtering a 3 nm thick Ti film, (3) annealing the structure in vacuum while raising the temperature from room temperature to 400 °C in 30 min, maintaining this temperature for 30 min, and cooling it down to room temperature in 30 min. As shown in Supplementary Figure 11b, positive-photocurrent generation in the dual-plasmon device with Schottky contact is a burst-like transient process. After about 400 sec of optical illumination, we obtained complete decay of the positive photocurrent from the peak to the initial value, meaning that the long-term illumination does not induce photocurrent. This is because a local electric field prevents electron transfer from the AuNPs to the TiO_2 layer. The local electric field is formed due to the spilling-off of the hot electrons from the AuNP. On the other hand, as shown in Supplementary Figure 11d, the decay time of the positive photocurrent was longer with the Ohmic contact. This observation reveals that the plasmonically induced charges were accumulated at the AuNP/TiO_2 and Au film/TiO_2 interfaces. In other words, it can be inferred that the charging-discharging effect is responsible for formation of the burst-like feature in the photocurrent measurements. To further elucidate the rectifying nature of contacts, I-V characteristics of the two devices are presented in Supplementary Figure 12. I-V measurements are carried out under the non-illumination condition. As shown in Supplementary Figure 12, the device with Schottky contact shows more rectifying characteristics than the device with Ohmic contact, supporting the concept of insertion of the Ti layer to be changing the potential barrier across the device. The offset of the current under zero-bias condition is attributed to the formation of an ultra-thin space-charge layer at the TiO_2/water interface.

Supplementary Note 8: Fabrication of the dual-plasmon device for the proof-of-concept demonstration

For the proof-of-concept demonstration shown in Supplementary Movie 2, we performed PEC measurements in a two-electrode setting without the reference electrode (in which the counter and reference pins were connected) by using the dual-plasmon device as a working electrode and a 2 nm thick Pt film on a FTO layer as the counter electrode as shown in Supplementary Figure 13. The Pt film was fabricated by sputtering Pt onto a FTO/glass supporting substrate with a sputtering machine (ULVAC SH250). A 1.5 cm wide, 1 mm thick-bunched Teflon plate was used as a spacer between the two electrodes. The Teflon plate was securely sealed to the active layer and Pt electrode by a 100 µm thick polymer melting foil (Peccell Ltd., Japan) by heating it in a 110 °C hot plate under a certain pressure for 30 min. The 0.1M $KClO_4$ electrolyte solution was filled into the space formed by the Teflon plate and the two electrodes by injecting it via two holes (carved in the Teflon plate). Finally, the adjacent edges of the device were sealed by waterproof superglue.

SUPPLEMENTARY REFERENCES

1. Mukherjee, B., Wilson, W. & Subramanian, (R.) V. TiO_2 nanotube (T_NT) surface treatment revisited: Implications of ZnO, $TiCl_4$, and H_2O_2 treatment on the photoelectrochemical properties of T_NT and T_NT-CdSe. *Nanoscale* **5**, 269-274 (2013).
2. Pu, Y.-C., Wang, G., Chang, K.-D., Ling, Y., Lin, Y.-K., Fitzmorris, B. C., Liu, C.-M., Lu, X., Tong, Y., Zhang, J. Z., Hsu, Y.-J. & Li, Y. Au nanostructure-decorated TiO_2 nanowires exhibiting photoactivity across entire UV-visible region for photoelectrochemical water splitting. *Nano Letters* **13**, 3817-3823 (2013).
3. Johnson, P. & Christy, R. Optical constants of noble metals. *Physical Review B* **6**, 4730–4739 (1972).
4. http://www.sspectra.com/sopra.html (data of TiO_2 B). Date of access: August 23, 2016.
5. Moreau, A., Ciraci, C., Mock, J. J., Hill, R. T., Wang, Q., Wiley, B. J., Chilkoti, A. & Smith, D. R. Controlled-reflectance surfaces with film-coupled colloidal nanoantennas. *Nature* **492**, 86-89 (2012).
6. Read, T., Olkhov, V. R., & Shaw, A. M. Measurement of the localised plasmon penetration depth for gold nanoparticles using a non-invasive bio-stacking method. *Physical Chemistry Chemical Physics* **15**, 6122 (2013).
7. Novotny, L. Effective wavelength scaling for optical antennas. *Physical Review Letters* **98**, 266802 (2007).
8. Link, S. & El-Sayed, M. A. Spectral properties and relaxation dynamics of surface plasmon electronic oscillations in gold and silver nanodots and nanorods. *The Journal of Physical Chemistry B* **103**, 8410-8426 (1999).
9. Chen, H., Liu, G. & Wang, L. Switched photocurrent direction in Au/TiO_2 bilayer thin films. *Scientific Report* **5,** 10852 (2015).
10. Zheng, B. Y., Zhao, H., Manjavacas, A., McClain, M., Nordlander, P. & Halas, N. J. Distinguishing between plasmon-induced and photoexcited carriers in a device geometry. *Nature Communications* **6**, 7797 (2015).
