# Supplementary material for: Interplay of hot electrons from localized and propagating plasmons
Source: Nat Commun. 2017 Oct 3;8:771. doi: 10.1038/s41467-017-00815-x (PMC5626744; doi:10.1038/s41467-017-00815-x)
Supplement: Supplementary file 3 — Description of Additional Supplementary Files [file 41467_2017_815_MOESM3_ESM.pdf]

File Name: Supplementary Movie 1

Description: Animated Movie showing the functionality of the dual-plasmon device

File Name: Supplementary Movie 2

Description: Demonstration of the wavelength-controlled polarity-switchable photoconductance.
